# Projected climate change impact on a coastal sea—As significant as all current pressures combined

**DOI:** 10.1111/gcb.16312

**Published:** 2022-07-06

**Authors:** Iréne Wåhlström, Linus Hammar, Duncan Hume, Jonas Pålsson, Elin Almroth‐Rosell, Christian Dieterich, Lars Arneborg, Matthias Gröger, Martin Mattsson, Lovisa Zillén Snowball, Gustav Kågesten, Oscar Törnqvist, Emilie Breviere, Sandra‐Esther Brunnabend, Per R. Jonsson

**Affiliations:** ^1^ Research Department Swedish Meteorological and Hydrological Institute Norrköping Sweden; ^2^ Octopus Ink Research & Analysis Lysekil Sweden; ^3^ Geological Survey of Sweden Uppsala Sweden; ^4^ Kelonia AB Göteborg Sweden; ^5^ Department of Physical Oceanography and Instrumentation Leibniz‐Institute for Baltic Sea Research (IOW) Rostock Germany; ^6^ Mountainlake Göteborg Sweden; ^7^ Department of Marine Sciences, Tjärnö Marine Laboratory University of Gothenburg Strömstad Sweden

**Keywords:** Baltic Sea, climate change, cumulative effects, Delphi method, ecosystem‐based management, marine spatial planning, multiple stressors, Symphony

## Abstract

Climate change influences the ocean's physical and biogeochemical conditions, causing additional pressures on marine environments and ecosystems, now and in the future. Such changes occur in environments that already today suffer under pressures from, for example, eutrophication, pollution, shipping, and more. We demonstrate how to implement climate change into regional marine spatial planning by introducing data of future temperature, salinity, and sea ice cover from regional ocean climate model projections to an existing cumulative impact model. This makes it possible to assess climate change impact in relation to pre‐existing cumulative impact from current human activities. Results indicate that end‐of‐century projected climate change alone is a threat of the same magnitude as the combination of all current pressures to the marine environment. These findings give marine planners and policymakers forewarning on how future climate change may impact marine ecosystems, across space, emission scenarios, and in relation to other pressures.

## Graphical abstract

Our seas are under high pressures from human activities and climate change. The results from this study indicate that end‐of‐century projected climate change alone is a threat of the same magnitude as the combination of all current pressures to the marine environment. We developed methods to incorporate climate change into a cumulative impact model and to bridge some of the knowledge gaps on how sensitive different marine ecosystems are to climate change. These methods can be implemented in other regional seas. It will also facilitate for policymakers to undertake mitigation and adaptation measures and conduct marine spatial planning.

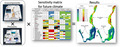

## 1 INTRODUCTION

Holistic perspectives are necessary for addressing the complex challenges of sustainable use of our seas. Marine ecosystems are affected by numerous anthropogenic activities where fisheries, pollution, and eutrophication are responsible for much of marine degradation at scale while, for example, shipping, military, recreation, and coastal development contribute locally (Andersen et al., 2015; Bergström et al., 2019; Halpern et al., 2015; Hammar et al., 2020). In recent years, environmental changes caused by global climate change have become additional threats to marine ecosystems and caused significant impact in parts of the world (Hoegh‐Guldberg & Bruno, 2010). These climate change pressures and their added impact to marine ecosystems are expected to increase dramatically toward the end of this century (Henson et al., 2017; Perry et al., 2020). Despite this risk, climate change projections are rarely systematically incorporated with marine spatial planning (MSP) although this is an instrument for long‐term strategic planning (Santos et al., 2020). It is essential that these pressures are adequately accounted for within decision support tools for marine planning, management and policy, such as models for cumulative impact assessment.

Cumulative impacts can be interpreted and addressed through different methodological approaches (Korpinen & Andersen, 2016; Stelzenmuller et al., 2018). Within area‐based management, it is particularly useful to represent the effect of combined pressures on maps. Halpern et al. (2008) introduced a method of mapping cumulative impacts from human activities on marine ecosystems. This method has been commonly used within regional assessments and in support of MSP (Bergström et al., 2019; Gissi et al., 2017; Hammar et al., 2020; Korpinen et al., 2021; Korpinen & Andersen, 2016). Mapping of cumulative impacts allows the assessor, and consequently managers and policymakers, to gain a graphic overview of which areas are more affected than others and how much each human activity contributes proportionally to the cumulative impact in each area.

There are several challenges with including climate change into models for cumulative impacts. In this study, climate change alterations are treated as pressures, on the same level as, for example, contaminants or noise from human activities. One major challenge is to meaningfully account for uncertainties of anticipated climate change in temporally static models. Uncertainties are inevitably large, both regarding emission scenarios and within the chosen physical models used to project the changes in the local and regional physiochemical marine environment.

Another difficulty is how to assess the sensitivity of different ecosystems and organisms to climate change pressures. Some general sensitivity scores for climate change alterations have earlier been acquired from expert surveys (Halpern et al., 2015; Korpinen et al., 2021). But in order to avoid exaggeration of today's climate change, when worst changes are yet to come, expert surveys and subsequent spatial models need to be precise on the climate change under question (e.g., “with surface water temperature rise we mean +3°C annual average”).

**FIGURE 1:**
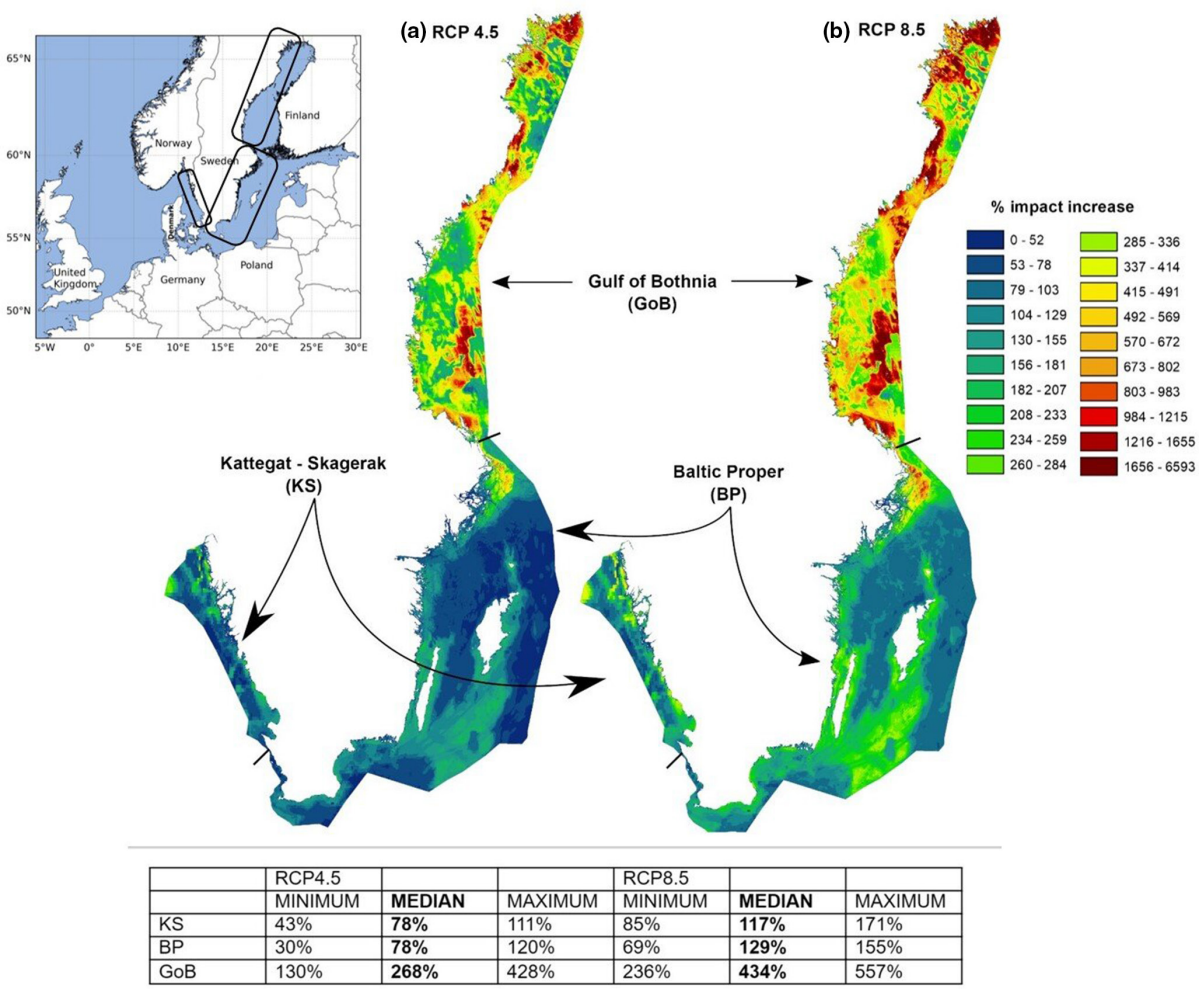
Impact maps for the analyzed area. Impact maps for the Kattegat‐Skagerrak, Baltic Proper, and the Gulf of Bothnia showing the percentage change between the baseline and the MEDIAN model ensemble for RCP4.5 (a) and RCP8.5 (b). Embedded text boxes denote change of cumulative impact per area (over the calculated anthropogenic environmental impact from human activities), including values for MINIMUM, MEDIAN, and MAXIMUM model ensembles. The impact maps are based on the geographical distribution of ecosystem components and pressures, plus the sensitivity scores. The small map shows the Northern Europe and approximately the three analyzed areas covering the Swedish territorial water and EEZ. Note, the three different areas have been analyzed separately and are not directly comparable.

The study was performed in water surrounding Sweden, in Northern Europe, as an example of how climate change can be implemented in a tool for regional MSP. These are eutrophic seas covering the Swedish territorial waters and Exclusive Economic Zone (EEZ), from northern Gulf of Bothnia, south to the central Baltic Proper and northwest through Kattegat to Skagerrak (Figure 1). The study area is composed of several shallow and narrow straits reducing water exchange with the North Sea. It presents a natural salinity gradient, with almost fresh water in the north and marine waters in the south‐west, along which marine ecosystems vary. Therefore, the analyses were performed for three distinct geographical areas: Kattegat‐Skagerrak, Baltic Proper, and the Gulf of Bothnia.

In this study, we aim to systematically integrate projected climate change pressures with other, better known, human disturbances to marine ecosystems. This is to facilitate for assessors and managers to conceive the magnitude of climate change and develop appropriate measures for adaptation. We introduce end‐of‐century climate change as additional pressures to the pre‐existing cumulative impact model, Symphony, utilized within Swedish MSP (Hammar et al., 2020) and based on previous work (Halpern et al., 2008). We use multiple ensembles of global climate models, downscaled by a regional coupled atmosphere–ocean model for the Baltic Sea and North Sea, accounting for the RCP4.5 and 8.5 emission scenarios. For the first time, a novel method to obtain climate change sensitivities was applied, based on detailed questionnaires and Delphi method discussions between subject matter experts. Results illustrate how threats from climate change compare to threats from other currently prevailing pressures, and how climate change impacts may vary between locations.

## 2 METHODS

### 2.1 Basic cumulative impact model

Cumulative impact refers to the relative effect of combined pressures from different kinds of human activities on components of marine ecosystems and was calculated using the model framework developed earlier (Halpern et al., 2008). In each pixel in a geographic grid, an impact index (*I*_sum_) is calculated from the combined multiplication of values representing valuable ecosystem components (*E*), pressure intensities (*P*), and the specific sensitivity (*S*) of every ecosystem component to every pressure, according to Equation (1):

(1) $$I_{\mathrm{sum}}=\sum_{i=1}^{n}\sum_{j=1}^{m}P_{i}\times E_{j}\times S_{i,j}$$

We adopted the pre‐existing model, Symphony, including 32 ecosystem components (e.g., benthic habitats, populations of fish, marine mammals); 39 pressures (e.g., fishing, eutrophication, shipping); and non‐climate change sensitivity scores generated from previously collected expert panel questionnaires (Hammar et al., 2020).

### 2.2 Climate change pressure data

Modeled climate change was added to the Symphony model as pressures denoting the difference between two 30‐year averages for a historical reference period (1976–2005) and an end‐of‐century period (2070–2099). The changes were calculated for five parameters; winter averaged sea‐ice cover (percentage of lost sea‐ice cover days, November–April), annual averaged surface and bottom water salinity, and summer averaged surface and bottom water temperature (May–August). The summer temperature was chosen as it is most important for biological activities and because marine organisms of the region are sensitive to the higher spectrum of temperature which occurs during summer.

The climate projections are based on the assumptions used in the 2013 Intergovernmental Panel on Climate Change Representative Concentration Pathway (RCP) scenarios for greenhouse gas forcing (IPCC, 2013). To account for uncertainties in the climate projections, an ensemble of five global climate models was downscaled using the high‐resolution‐ocean–sea‐ice–atmosphere model RCA4–NEMO (Gröger et al., 2019). Two scenarios, RCP4.5 and RCP8.5 (Moss et al., 2010), were considered representing an intermediate and an extreme scenario.

To obtain an uncertainty measure of the two climate emission scenarios and the five climate change parameters, the ensemble minimum, median, and maximum values were calculated for each of the two emission scenarios. These were interpreted as a range of climate change pressures in the cumulative impact calculations. MINIMUM corresponds to the lowest model ensemble values, MEDIAN to the median ensemble values, and MAXIMUM to the highest ensemble values (Figures S1–S5).

### 2.3 Sensitivity scores for climate change

It is not well known how future changes to temperature, salinity, and sea‐ice cover will influence different ecosystem components. These sensitivities are key elements to the model used. A panel consisting of subject matter experts was assembled and divided into four groups: (1) benthic and pelagic habitats, (2) seabirds, (3) marine mammals, (4) fish. Panel groups were assigned to assess the sensitivity of their corresponding ecosystem components species‐by‐species, to each of the five climate change pressures. The degree of change was specified for each pressure so experts could assess response levels, sensitivities, with best possible accuracy: +3°C/+4°C for bottom/surface water temperature; −2 PSU/−1.5 PSU for bottom/surface water salinity; 40% decrease of sea‐ice cover. The degrees of change were based on the climate ensemble mean outcome for RCP8.5 and represented the corresponding maximum grid value for climate change pressures within the Swedish EEZ.

A Delphi method (Dalkey & Helmer, 1963) approach was used to refine the results from the expert panel groups. First, each expert answered all questions individually using online forms. Second, each expert group met virtually to consider the anonymized results from the individual exercises. They discussed and then anonymously voted for the most applicable sensitivity score for each pressure and ecosystem component combination. The benefit of this method was that it reduced uncertainty as experts shared with each other their expertise, literature findings, and causal linkages behind assessments, to conform their interpretation of the task and individual assessments.

Six sensitivity rating categories were explicitly defined, ranging from 0 (no effect) to 1 (permanent loss, very high mortality), using the same wordings as for the previously addressed nonclimate change‐related pressures (Hammar et al., 2020). Experts were requested to denote possible differences in sensitivity between the three geographical areas. Once the sensitivity ratings were collected, we used the mode values from each group to assign sensitivity scores, representing how sensitive different ecosystem components are to each of the covered climate change pressures in the three areas. Following the general method, only positive sensitivity scores were assigned. That is, only degrading environmental effects were considered. In the rare case of an ecosystem component being expected to benefit from the climate change pressure, the sensitivity score was set to 0. The failure to include possibly beneficial effects when calculating the cumulative impact is a limitation to the method.

## 3 RESULTS

### 3.1 Climate change sensitivities

The results from the expert panel for the climate change pressure sensitivity scores are presented in Table 1. Across ecosystem components, temperature rise and bottom water salinity reduction were considered the most harmful climate change pressures. Surface water salinity reduction and sea‐ice cover loss were in most, but not all, cases considered benign.

**TABLE 1:** Climate change pressures' sensitivity scores based on the results of the expert panel survey using the Delphi method. Scores represent the mode values set by experts within panel groups, each addressing one of the four groups of ecosystem components. Experts addressed differences between the three areas: Kattegat‐Skagerrak (KS); Baltic Proper (BP); Gulf of Bothnia (GoB). All ecosystem components do not exist in every area (−). Each ecosystem component may only be sensitive to either bottom or surface water changes (to avoid double counting in the cumulative impact model). Levels of change, denoted in the top row, are based on ensemble mean grid maximum values for RCP8.5.

Sensitivity scores		Temp. Bottom +3°C			Temp. Surface +4°C			Salinity bottom −2 PSU			Salinity surface −1.5 PSU			Ice cover −40% of time		
Ecosystem components		KS	BP	GoB	KS	BP	GoB	KS	BP	GoB	KS	BP	GoB	KS	BP	GoB
Birds	Coastal birds				0.2	0.2	0.2				0	0	0	0.2	0.2	0.2
	Seabirds coastal wintering				0.2	0.2	0.2				0	0	0	0	0	0
	Seabirds offshore wintering				0.2	0.2	0.2				0	0	0	0	0	0
Mammals	Porpoise North Sea population				0.2	—	—				0	—	—	0	—	—
	Porpoise Baltic population				—	0.2	0.2				—	0	0	—	0	0
	Porpoise Belt population				0.2	0.2	—				0	0	—	0	0	—
	Gray seal				0	0	0				0	0	0	0.4	0.4	0.4
	Harbor seal				0	0	—				0.2	0.2	—	0	0	—
	Ringed seal				—	—	0.4				—	—	0	—	—	0.8
Fish	Cod	0.8	0.6	0.6				0.2	0.4	0.6				0	0	0
	Herring	0.4	0.2	0.2				0	0.2	0.4				0	0	0
	Sprat	0	0	0				0.2	0.4	0.6				0	0	0
	Vendace	—	—	0.6				—	—	0.2				—	—	0.4
	Fish spawning	0.6	0.4	0.4				0.4	0.6	0.8				0.2	0.2	0.4
	River mouth fish				0.6	0.6	0.6				0	0	0	0.2	0.2	0.4
	Eel migration	0	0	0				0	0	0.2				0	0	0
Habitats and structure forming organisms	Mussel reef	0.6	0.6	0.6				0.2	0.8	0.9				0	0	0
	Deep reef	1	1	1				1	1	1				0	0	0
	*Haploops* reef	1	1	—				1	1	—				0	0	—
	Artificial reef	0.6	0.6	0.6				0.2	0.6	0.9				0	0	0
	Angiosperms				0.8	0.8	0.8				0	0.2	0	0	0	0
	Shoreline				0.8	0.8	0.8				0.2	0.2	0.2	0.2	0.2	0.2
	Hard bottom photic				0.8	0.8	0.8				0.5	0.4	0.9	0	0	0
	Hard bottom aphotic	0.8	0.6	0.4				0.4	0.6	0.2				0	0	0
	Hard bottom deep	1	0.6	0.4				0.8	0.2	0.2				0	0	0
	Transport bottom photic				0.8	0.8	0.8				0.4	0.4	0.8	0	0	0
	Transport bottom aphotic	0.8	0.6	0.4				0.4	0.6	0.2				0	0	0
	Transport bottom deep	0.9	0.6	0.4				0.6	0.2	0.1				0	0	0

	Soft bottom photic				0.8	0.8	0.8				0.4	0.4	0.2	0.2	0.2	0.2
	Soft bottom aphotic	0.8	0.6	0.6				0.7	0.2	0.2				0	0	0
	Soft bottom deep	0.8	0.6	0.6				0.8	0.4	0.2				0	0	0
Pelagic plankton community				0.4	0.4	0.4				0.2	0.2	0.2	0.2	0.2	0.2

Sensitivity categories	
0	No effect OR Negligible effect
0.1–0.2	Slight disturbance/stress with consequence only from a cumulative perspective
0.3–0.4	Disturbance/stress with influence on survival or reproduction rate
0.5–0.6	Disturbance/stress with strong influence on survival or reproduction rate OR Occasional immediate destruction/mortality
0.7–0.8	Frequent immediate destruction/mortality
0.9–1	Permanent loss or very high mortality

In the Kattegat‐Skagerrak area, the habitats sensitivity to salinity reduction is higher than in the other two areas. There is also high sensitivity of ecosystem to the increased bottom water temperature.

Overall, benthic habitats, sessile organisms, and fish were considered more sensitive to temperature and salinity changes, while seabirds and marine mammals, being air‐breathing and mobile, attained comparatively low sensitivities.

### 3.2 Climate change escalation of cumulative impact

**FIGURE 2:**
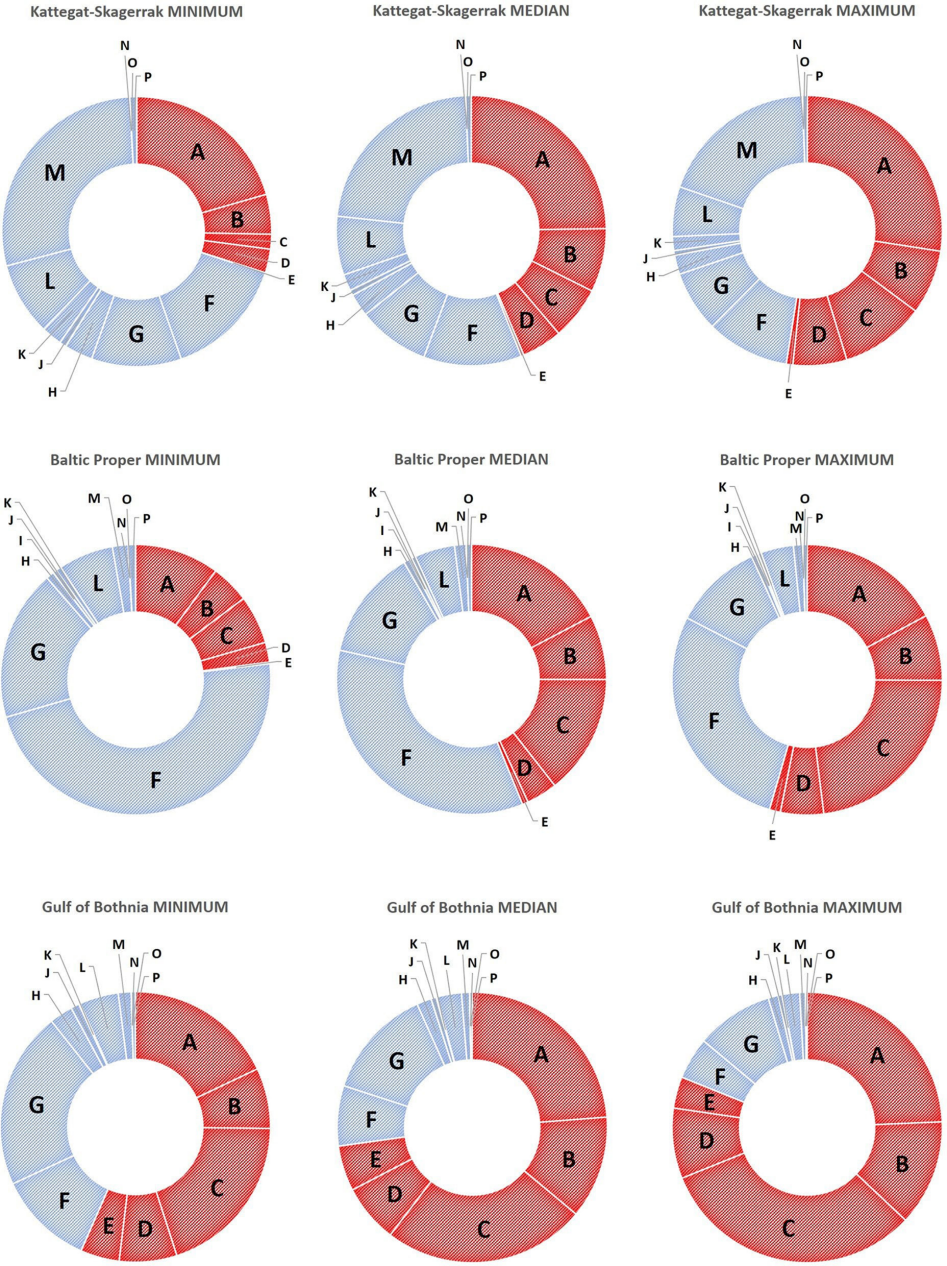
Climate change impact for RCP4.5 relative to other environmental pressures. Proportional impact contributions from modeled climate change pressures (red) and other impact sources (blue) to the marine environment in the Kattegat‐Skagerrak, Baltic Proper, and the Gulf of Bothnia, for the MINIMUM, MEDIAN, and MAXIMUM in RCP4.5. Pressures from human activities have been categorized into the following source categories: A—Climate change‐driven temperature rise in bottom water; B—Climate change‐driven temperature rise in surface water; C—Climate change‐driven salinity reduction in bottom water; D—Climate change‐driven salinity reduction in surface water; E—Climate change‐driven ice cover reduction; F—Eutrophication; G—General pollution (oil spill from wreck, heavy metals (background, mine dump), synthetic toxins (background), dump of toxic munition); H—Industry (heavy metals [fiber bank, mercury dump], synthetic toxins from industries and in harbor); I—Sand extraction; J—Coastal development; K—Recreation; L—Shipping; M—Fisheries: N—Aquaculture; O—Energy; P—Military defense.

The sensitivities presented above are important components of the spatial model of cumulative impact, which also includes spatial distributions of ecosystem components and pressures from climate change as well as other anthropogenic sources. In the RCP4.5, the MEDIAN level of the climate change pressure results in 78% additional impact to the current cumulative impact in the Kattegat‐Skagerrak and Baltic Proper areas, and 268% additional impact to the Gulf of Bothnia (Figures 1a and 2). Even at the MINIMUM level, climate change adds a remarkable impact compared to the situation of today (Kattegat‐Skagerrak +43%; Baltic Proper +30%; Gulf of Bothnia +130%). The MAXIMUM level means a doubling compared to existing cumulative pressures for the Kattegat‐Skagerrak and Baltic Proper areas, while a fourfold increase is expected in the Gulf of Bothnia (Figures 1a and 2).

**FIGURE 3:**
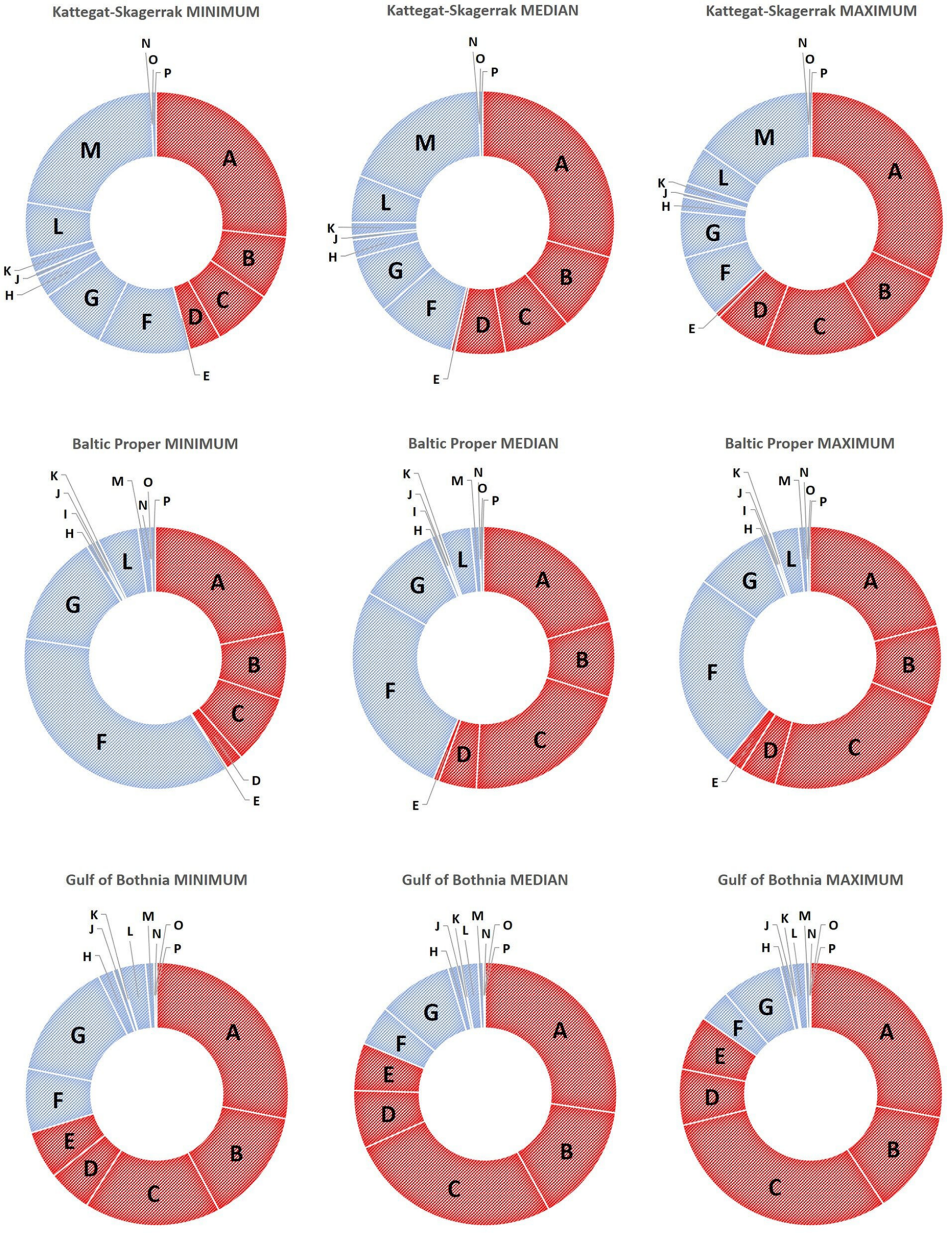
Climate change impact for RCP8.5 relative to other environmental pressures. Proportional impact contributions from modeled climate change pressures (red) and other impact sources (blue) to the marine environment in the Kattegat‐Skagerrak, Baltic Proper, and the Gulf of Bothnia, for the MINIMUM, MEDIAN, and MAXIMUM in RCP8.5. Pressures from human activities have been categorized into the following source categories: A—Climate change‐driven temperature rise in bottom water; B—Climate change‐driven temperature rise in surface water; C—Climate change‐driven salinity reduction in bottom water; D—Climate change‐driven salinity reduction in surface water; E—Climate change‐driven ice cover reduction; F—Eutrophication; G—General pollution (oil spill from wreck, heavy metals (background, mine dump), synthetic toxins (background), dump of toxic munition); H—Industry (heavy metals [fiber bank, mercury dump], synthetic toxins from industries and in harbor); I—Sand extraction; J—Coastal development; K—Recreation; L—Shipping; M—Fisheries: N—Aquaculture; O—Energy; P—Military defense.

In the RCP8.5, the MEDIAN level of climate change pressure already indicates a doubling of impact in the Kattegat‐Skagerrak and Baltic Proper areas and a fourfold increase in the Gulf of Bothnia (Figures 1b and 3).

For both emission scenarios, results point at bottom water temperature rise constituting the largest climate change threat to the Kattegat‐Skagerrak area, while the other two areas are equally threatened by temperature and salinity changes, with salinity reduction being the largest threat under the MAXIMUM climate pressure (Figures 2 and 3).

Under RCP4.5, the highest relative increase of cumulative impact occurs in some of the most pristine areas. These become areas of particular concern: north‐western Kattegat‐Skagerrak; central and northernmost Baltic Proper; the deep of Bothnian Sea and the three shallow sills in southern, central, and northern Gulf of Bothnia (Figure 1a). At the RCP8.5, these areas of concern expand and new areas emerge, such as coastal stretches of the central and southern Kattegat‐Skagerrak area (Figure 1b).

## 4 DISCUSSION

This study incorporates results from models of future climate change into a spatial model of current human impact on Swedish seas. The temporal skewness (adding future pressures to a current state modes), together with the general ambiguity of both climate change models (Payne et al., 2016) and cumulative impact models (Gissi et al., 2017; Hodgson et al., 2019; Stock & Micheli, 2016), inevitably implies that several tiers of uncertainty are intrinsic to the results. Nevertheless, the underpinning models and methods are best practice and despite temporal skewness, scenario‐based cumulative impact models have proven valuable for management (Hammar et al., 2020). Given the importance of preventive actions to combat climate change challenges, this study may serve as a valuable hint of the magnitude of impending impacts.

The findings indicate that studied climate change pressures constitute paramount threats to marine ecosystems, with projected impacts being comparable to the combined impact of all other pressures of today. Because ocean warming and salinity changes permeate the environment across vast areas, the very preconditions for marine life may be altered. This will have fundamental implications for keystone species which live near their physiological tolerance limits (Vuorinen et al., 2015; Wåhlström et al., 2020).

The strong salinity gradient in the region influences the sensitivity to salinity changes. Fish and mussel reefs (reefs of *Mytilus edulis/trossulus*) are believed to be more sensitive to salinity reductions further north in the Baltic Sea, with highest sensitivity in the brackish Gulf of Bothnia where several species already live close to their salinity tolerance. On the contrary, when considering habitats instead of individual species, the sensitivity to salinity reduction is considered higher in the saline Kattegat‐Skagerrak, because the highly diverse marine ecosystems are not as tolerant to changes as the brackish water ecosystems of the Baltic Proper. However, the results from Meier et al. (2021) show that the projected salinity changes may be overestimated in the present study where the influence of sea level rise on saline inflows is not included, which would reduce the modeled impact from salinity change in some areas. This points at the need for continuous input from marine climate research to impact assessment and management response.

Another difference is the high sensitivity of ecosystem components in Kattegat‐Skagerrak to bottom water temperature rise. The marine deep‐water fauna is not well adapted to fluctuations. Habitats and sessile organisms, unable to avoid fluctuations by moving, were deemed the most sensitive here. According to the sensitivity scores (Table 1), the changes in ice cover and surface freshening have less influence on the surface compare to the benthic ecosystem. However, these surface changes might have indirect effect on the ecosystem as they can alter the algal type and increase the primary productivity, both prolong the growth season and increase growth rate. These results are not surprising from an ecological viewpoint, but must be carefully represented in spatial models that span across natural environmental.

The modeled impact contribution from climate change varies greatly between the three studied areas. Partly, this reflects different environmental and oceanographic characteristics of these areas. Another important difference is that current human impact strongly varies between the areas, with much less ongoing pressure in the northern Gulf of Bothnia. Here, the added climate change‐related impact becomes proportionally higher, causing ecosystems to experience a higher total change of exposure to stress, although the absolute climate‐driven impact may not be worse than elsewhere (this varies between locations).

It should be noted that cumulative impact models do not capture past impacts, for example, historically lost or deprived species (Hammar et al., 2020). The results should not be interpreted as to depreciate the importance of past and current pressures, but to raise the warning that climate change may be an equally serious threat within the tangible future.

Decision makers are urged to incorporate this aspect in plans and measures, such as MSP and action plans. The Swedish MSP identifies possible climate refugia (SwAM, 2022) and climate adaptation plans are developed nationally and locally. This study highlights the importance of maintaining and developing such work, also internationally. The magnitude of possible change, even for low‐risk scenarios, calls for preparedness for fundamental ecosystem changes. Over the next decades, we need methods for meaningful conservation in a rapidly changing climate as well as alternative harvest and management strategies, while continuing to reduce the cumulative impact from human activities.

This study provides an overview of climate change effects incorporated into a cumulative impact model. It highlights areas of particular concern and adds value to the method development within cumulative impact models, through the Delphi method for developing sensitivity scores. Even though the sensitivity scores provided may have direct value for some applications of the model (Halpern et al., 2008), we argue that it is fundamental to continuously refine sensitivity scores from case to case when dealing with environmental change such as climate change. Previous research has compared sensitivity scoring based on expert panels and found only small differences between regions (Korpinen et al., 2021). We highlight that natural environmental gradients, as those present in the Baltic Sea, require intricate efforts on setting sensitivity scores, to account for organisms that inhabit environments close to their physiological tolerance limits.

## AUTHOR CONTRIBUTIONS

IW, LH, DH, JP, EAR, MG, LZS, GK, and PRJ conducted the conceptual development of the study. MG, DH, CD, LA, and OT lead the implementation of climate data and uncertainties. LH and IW drafted the manuscript with substantial contribution from DH and JP. DH, MM, and LH produced the figures. JP and LH developed and performed the expert panel analysis for sensitivity scores. MM implemented climate data with the Symphony model. All authors contributed substantially with important intellectual content and editing of the manuscript. All authors read and approved the submitted manuscript version.

## CONFLICT OF INTEREST

The authors declare no competing interests.

## Supporting information


Appendix S1

## Data Availability

The data supporting the findings of this study are openly available in the Swedish National Data Service (SND) at https://doi.org/10.5878/gwas‐0254.
