# Supplementary material for: Projected climate change impact on a coastal sea—As significant as all current pressures combined
Source: Glob Chang Biol. 2022 Jul 6;28(17):5310–9. doi: 10.1111/gcb.16312 (PMC9541661; doi:10.1111/gcb.16312)
Supplement: Supplementary file 1 — Appendix S1 [file GCB-28-5310-s001.pdf]

Supplementary information

Projected climate change impact on a coastal sea is comparable to current pressures combined

Iréne Wåhlström[1*], Linus Hammar, Duncan Hume[2], Jonas Pålsson, Elin Almroth-Rosell[1], Christian Dieterich[1,†], Lars Arneborg[1], Matthias Gröger[1,3], Martin Mattsson[4], Lovisa Zillén Snowball[2], Gustav Kågesten[2], Oscar Törnqvist[2], Emilie Breviere[1], Sandra-Esther Brunnabend[1], Per R. Jonsson[5]

Affiliations:

[1] Swedish Meteorological and Hydrological Institute, Norrköping, Sweden

[2] Geological Survey of Sweden, Uppsala, Sweden

[3] Leibniz-Institute for Baltic Sea Research (IOW), Rostock, Germany

[4] Medins Havs och Vattenkonsulter AB, Mölnlycke, Sweden

[5] Department of Marine Sciences, Tjärnö Marine Laboratory, University of Gothenburg, Strömstad, Sweden

[†] Deceased

***Corresponding author:** E-mail: irene.wahlstrom@smhi.se

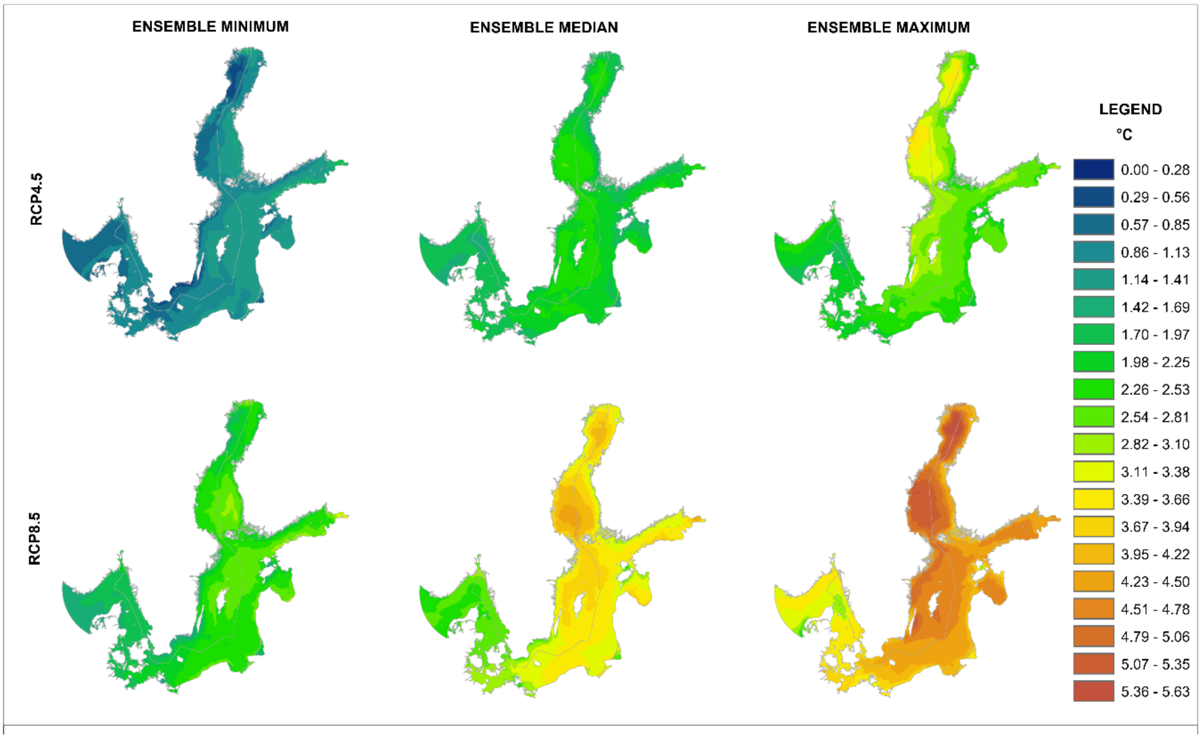

Supplementary Figure 1. Predicted change in average summer season (May-Aug) surface water temperature between 1976-2005 and 2070-2099 under two climate scenarios (RCP4.5 and 8.5) with the ensemble MINIMUM, MEDIAN and MAXIMUM for the five downscaled regional climate models.

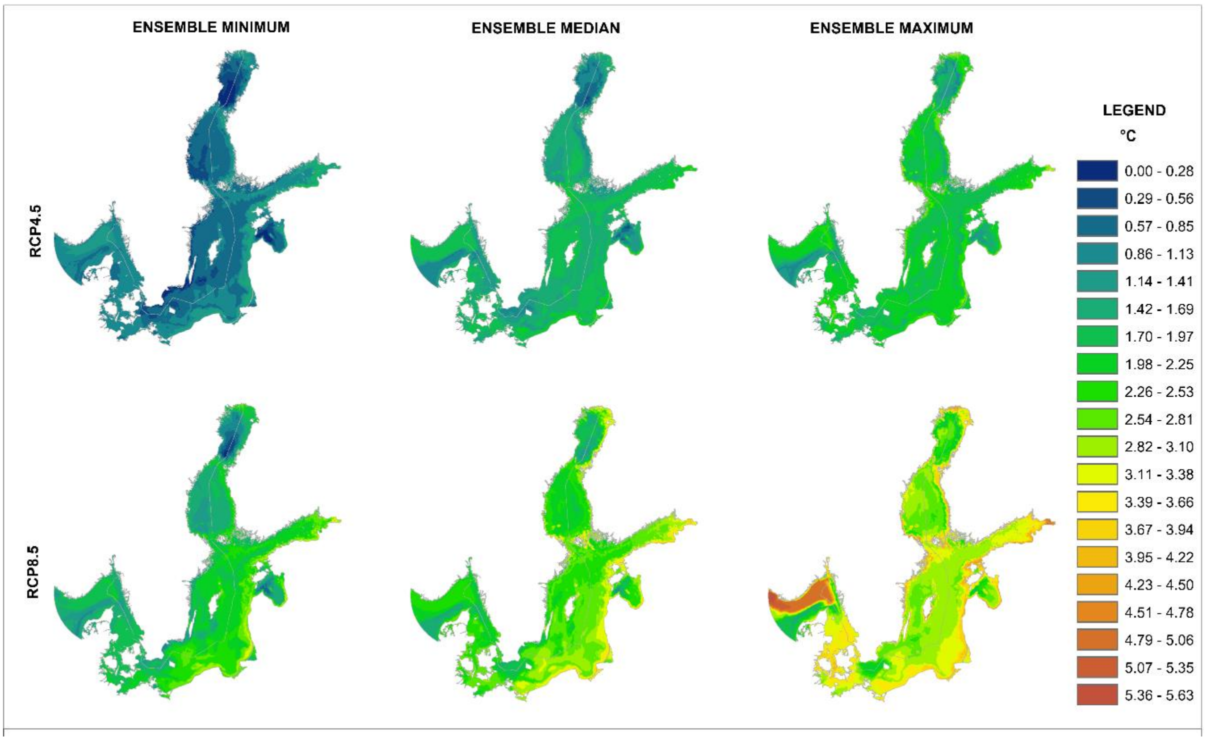

Supplementary Figure 2. Predicted change in average summer season (May-Aug) bottom water temperature between 1976-2005 and 2070-2099 under two climate scenarios (RCP4.5 and 8.5) with the ensemble MINIMUM, MEDIAN and MAXIMUM for the five downscaled regional climate models.

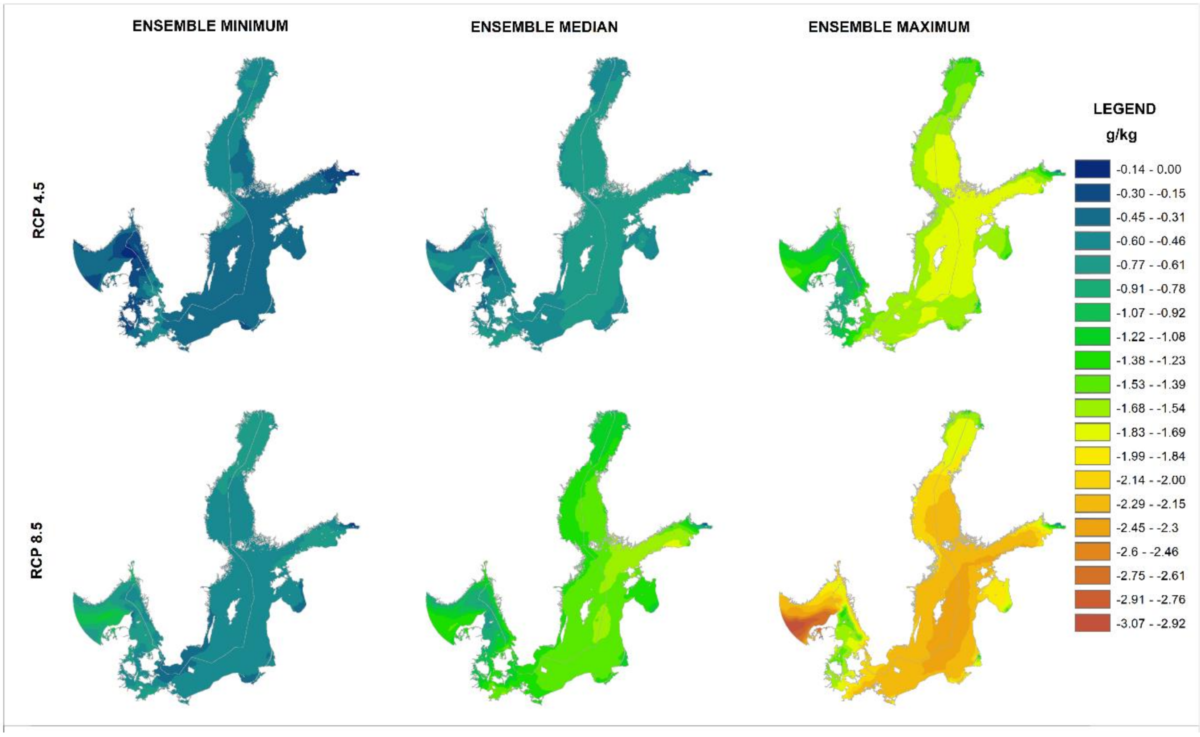

Supplementary Figure 3. Predicted change in average annual surface water salinity between 1976-2005 and 2070-2099 under two climate scenarios (RCP4.5 and 8.5) with the ensemble MINIMUM, MEDIAN and MAXIMUM for the five downscaled regional climate models.

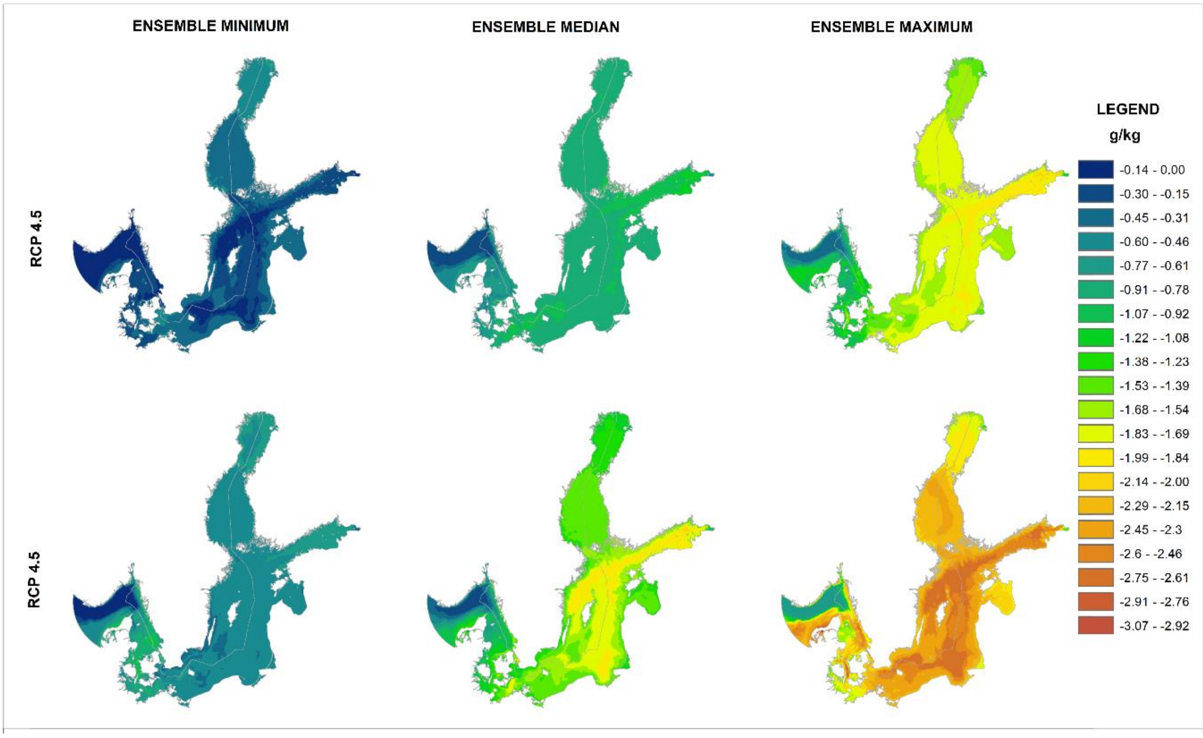

Supplementary Figure 4. Predicted change in average annual bottom water salinity between 1976-2005 and 2070-2099 under two climate scenarios (RCP4.5 and 8.5) with the ensemble MINIMUM, MEDIAN and MAXIMUM for the five downscaled regional climate models.

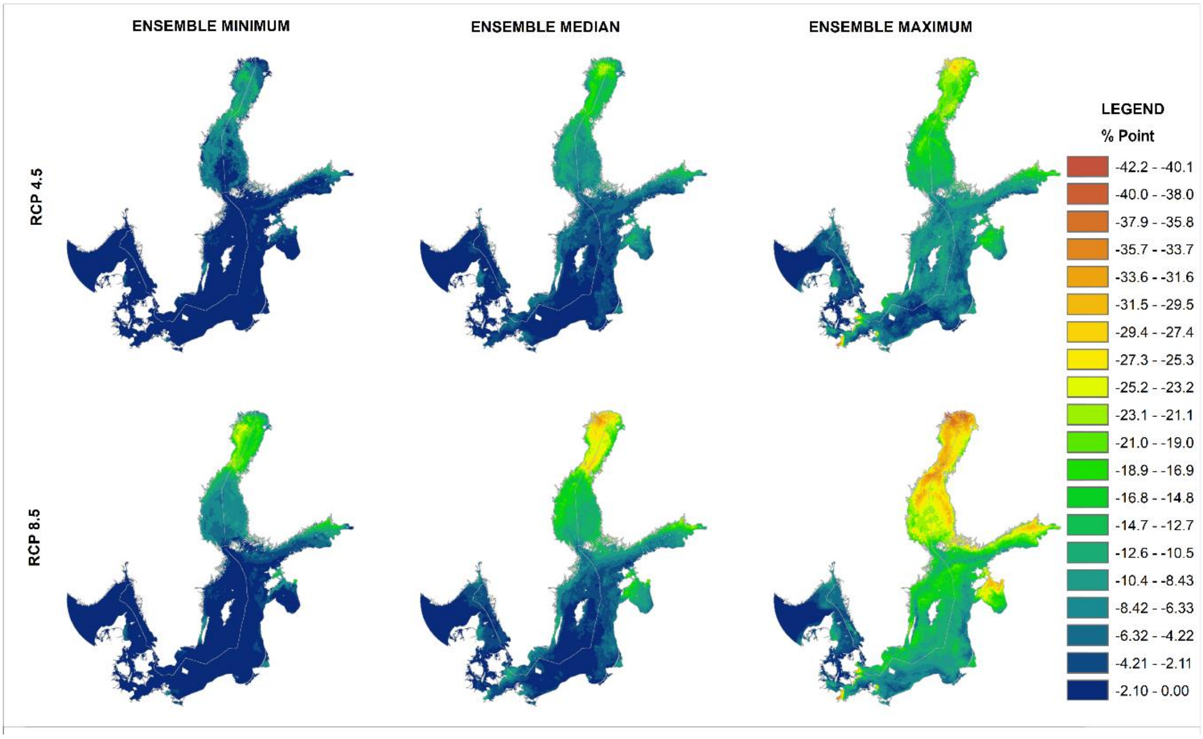

Supplementary Figure 5. Predicted change in average winter (Nov-April) sea ice coverage between 1976-2005 and 2070-2099 under two climate scenarios (RCP4.5 and 8.5) with the ensemble MINIMUM, MEDIAN and MAXIMUM for the five downscaled regional climate models.
